# *Lr67* and *Lr34* rust resistance genes have much in common – they confer broad spectrum resistance to multiple pathogens in wheat

**DOI:** 10.1186/1471-2229-13-96

**Published:** 2013-07-02

**Authors:** Wolfgang Spielmeyer, Rohit Mago, Colin Wellings, Michael Ayliffe

**Affiliations:** 1 CSIRO Plant Industry, GPO Box 1600, Canberra, ACT, 2601, Australia; 2 Plant Breeding Institute, University of Sydney, Private Bag 4011, Narellan, NSW, 2567, Australia

**Keywords:** Lr34, Lr67, Rust resistance, Mutants

## Abstract

**Background:**

Adult plant rust resistance genes *Lr67* and *Lr34* confer race non-specific resistance to multiple fungal pathogens of wheat. Induced, susceptible mutants were characterised for both genes.

**Results:**

Three categories of Lr34 mutants were identified that were either partial susceptible, fully susceptible or hyper-susceptible to stripe rust and leaf rust. The likely impact of the mutational change on the predicted Lr34 protein correlated with differences in response to rust infection. Four independent Lr67 mutants were recovered that were susceptible to stripe rust, leaf rust and stem rust pathogens, including one possible hyper-susceptible Lr67 mutant.

**Conclusions:**

Detailed study of Lr34 mutants revealed that subtle changes in resistance response to multiple pathogens were correlated with mutational changes in the predicted protein. Recovery of independent Lr67 mutants indicates that as for *Lr34*, a single gene at the *Lr67* locus is likely to confer resistance to multiple pathogens. The infection phenotypes of Lr67 mutants closely resembled that of Lr34 mutants.

## Background

Many rust resistance genes are known in wheat but only a few provide durable, race non-specific resistance to multiple pathogens [1]. The most widely deployed and best characterised of these genes, *Lr34,* confers durable, adult plant resistance (APR) to leaf rust caused by *Puccinia triticina*, stripe rust caused by *Puccinia striiformis* f.sp. *tritici* and powdery mildew caused by *Blumeria graminis*[2-5]. Lr34 resistance to each pathogen is partial while a second phenotype, so called leaf tip necrosis which involves necrosis of the tips of flag leaves, is also a characteristic of this gene [6]. More recently, *Lr67* was described which has similar characteristics to *Lr34* in that it also confers leaf tip necrosis and provides partial, broad spectrum, adult plant resistance to leaf rust and stripe rust [7,8]. Based on this phenotypic similarity *Lr67* is predicted to also provide durable rust resistance, although this remains inconclusive as the gene has not yet been deployed over long periods of time and across a diverse range of germplasm and environments.

When *Lr34* and *Lr67* were transferred from landraces into the leaf rust susceptible wheat variety ‘Thatcher’ , leaf rust resistant backcross lines also acquired resistance to stem rust (*P. graminis* f.sp. *tritici*) at both seedling and adult plant stages [9,10]. Seedling tests revealed that this stem rust resistance was probably race specific in contrast to the race non-specific responses of *Lr34* and *Lr67* to leaf rust and stripe rust. Further studies demonstrated that unlinked genetic loci interacted with *Lr34* to enable the expression of stem rust resistance in Thatcher and Thatcher derivatives [11-14].

In this study, the wheat line Thatcher was used as a common genetic background for the identification and comparison of mutant phenotypes in the *Lr34* and *Lr67* genes, respectively. Previous analysis of six *Lr34* mutants in the wheat line RL6058, a near-isogenic line of Thatcher with *Lr34*, confirmed that a single gene encoding a putative ATP-binding cassette transporter protein was responsible for *Lr34* resistance to leaf rust, stripe rust and stem rust [15]. We have isolated two additional RL6058 *Lr34* mutants and, in conjunction with the six previously identified mutants, undertaken a detailed characterisation of their response to stripe rust and stem rust infection under field conditions and in response to leaf rust at the seedling stage when grown under controlled conditions. Four Thatcher *Lr67* mutants were also identified which also showed a range of rust infection phenotypes closely resembling those of *Lr34* mutants, further supporting a mechanistic similarity between these two genes.

## Results

### Characterisation of Lr34 mutant phenotypes

Six sodium azide induced Lr34 mutants (2B, 2F, 2G, 3E, 4C, 4E) were previously reported that were susceptible to leaf rust, stripe rust and stem rust [15]. These mutants carried independent, single nucleotide substitutions resulting in either incorrect splicing of *Lr34* transcripts or changes in predicted amino acid sequence of the putative ABC transporter protein encoded by this gene (Table 1). These mutants also lost the leaf tip necrosis phenotype associated with the wild type gene. The response of these mutants to stripe rust infection was examined in three field seasons at Cobbitty, Australia. Hitherto undetected, subtle phenotypic differences between mutants were noted with mutant 4C showing a partial loss of resistance (60–70 MS) when compared with fully susceptible Thatcher (90S) and resistant wild type RL6058 (TR-5MR) lines. Mutant 2F developed more pustules on flag leaves earlier than Thatcher or other mutants while the remaining mutants were visually indistinguishable from Thatcher.

**Table 1:** Point mutations induced by sodium azide within genomic sequence of Lr34 gene in RL6058

**Mutant**	**mutation**	**Position of mutation (bp)**	**Effect on predicted protein**
2G	G to A	7167	amino acid 889 change G to D
4C	G to A	9108	amino acid 1030 change G to E
4G	G to A	3444	amino acid 228 change E to K
2B	C to T	5317	premature stop codon after amino acid 567
3E	G to A	738	splice site mutation at intron 3, premature stop
3F	G to A	9974	splice site mutation at intron 21, premature stop
4E	G to A	276	splice site mutation at intron 1, premature stop
2F	G to A	10495	splice site mutation at intron 22, partial deletion

**Figure 1:**
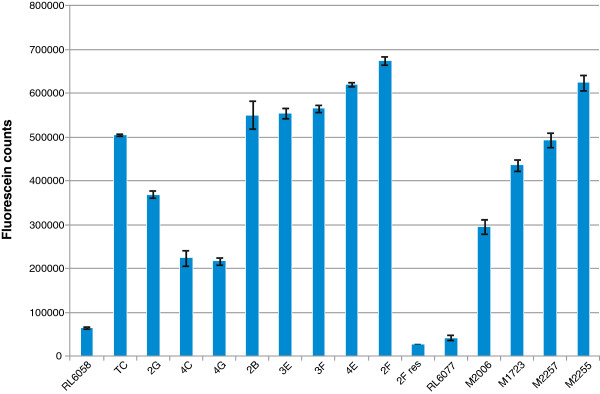
**Comparison of chitin levels in flag leaves of Lr34 and Lr67 mutants together with respective wild type RL6058 and RL6077 after infection with stripe rust.** Wheat lines are indicated on the X axis while relative chitin content is equivalent to the fluorescence values indicated on the Y axis in fluorescence units. Error bars show the standard error of mean of 4 technical replicates. For mutant 2F, a resistant sib line (2F res) was analysed from the same M2 family.

Two additional mutants (4G and 3F) were included in the third field season and flag leaf tissue was sampled from all eight mutants to quantify the stripe rust biomass accumulated at or near anthesis. Fungal biomass was quantified using the lectin wheat germ agglutinin (WGA), which specifically binds to chitin, conjugated to fluorescein isothiocyanate (FITC) and leaf tissue was processed such that the levels of fluorescence detected in each sample were equivalent to the amount of chitin present [16]. The results from the chitin assay confirmed field observations with mutant 4C showing an intermediate stripe rust resistance phenotype by accumulating 3.5 fold more chitin than the resistant wild type RL6058 but only half as much chitin as the susceptible Thatcher parent (Figure 1).

Similarly, mutant 4G, which showed partial susceptibility to stripe rust in the field, contained an intermediate level of chitin accumulation relative to the parental genotypes. Visual observations in the field did not distinguish mutant 2G from the susceptible Thatcher parent, however, a 30% reduction in chitin accumulation was observed which likely reflects the sensitivity of the chitin assay over empirical visual observations. In contrast, mutant 2F accumulated more chitin biomass than all other mutant lines and the susceptible Thatcher parent consistent with having an enhanced susceptibility to stripe rust infection. The remaining mutants accumulated chitin levels between the susceptible parent and the hyper-susceptible 2F mutant reflecting the increased variability in fungal infection between susceptible lines.

A loss of field resistance to stem rust was also observed in these mutant lines during two seasons in Australia. RL6058 was highly resistant (TR) while Thatcher was partially susceptible to stem rust (40–50 MRMS). Mutants were similar to Thatcher in their response to stem rust without apparent differences between mutants.

**Figure 2:**
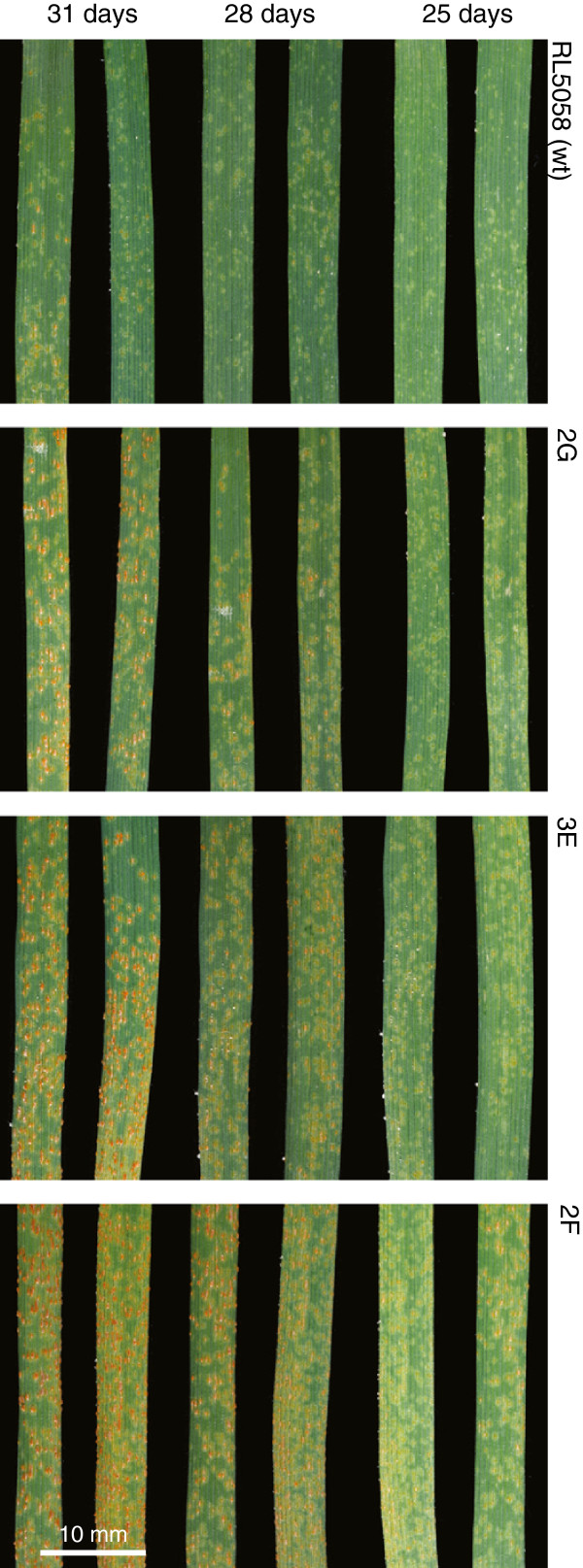
**Leaf rust infection on wheat first leaves at 25 days, 28 days and 31 days post inoculation at low temperature for RL6058 (*Lr34*) and three derived mutants 2G, 3E and 2F.** These three mutants show partial susceptibility, full susceptibility and hyper-susceptibility to leaf rust, respectively. Infection type of susceptible Thatcher was similar to mutant 3E and leaf rust infection of a resistant sib of mutant 2F was similar to RL6058 (not shown).

The response of these mutants to leaf rust infection was examined in seedlings grown under low temperature conditions [17]. The macroscopic rust infection phenotypes of mutants were observed at three time points and scored visually. Three mutants (2B, 3E and 4E) were fully susceptible and gave a similar response to the susceptible Thatcher parent (Figure 2). Two mutants, 2G and 4C, had an intermediate phenotype being more resistant than Thatcher but more susceptible than the wild type parent RL6058. Mutant 2F produced pustules earlier than Thatcher consistent with the hyper-susceptible phenotype of this mutant to stripe rust under field conditions.

To examine and quantify differences between the mutants in more detail, the infection process of leaf rust was studied microscopically after staining fungal structures with WGA-FITC [18]. Mutants were ranked in three separate experiments based on the area of infection hyphae which developed at randomly selected infection sites in first or second leaves 10 days post inoculation (Table 2). Mean leaf rust infection site areas were larger in 2G and 4C leaves compared to wild type RL6058, but smaller than Thatcher and the fully susceptible mutants 2B, 3E and 4E. These observations are consistent with the intermediate response of 2G and 4C recorded visually. Infection site areas were largest in mutant 2F and larger than in Thatcher in two experiments. The infection site areas of a resistant sib from the same M2 family were similar to wild type confirming that the observed phenotype of 2F was largely due to the mutation found within the *Lr34* gene and unlikely to be the result of accumulated background mutations (data not shown). Although there was variation between lines in rust infection site areas, the ranking of partial and fully susceptible categories remained the same across the three experiments. The lack of significant differences between mutant 2F and fully susceptible mutants was due to the large within-line variance observed for infection sites. Within-line variability probably also contributed to rank changes within the fully susceptible category.

**Table 2:** Mean infection site areas for leaf rust growth at 10 dpi on RL6058 (wt) and derived mutants 2G, 4C, 3E, 4E, 2B and 2F across three experiments

**Expt1**			**Expt2**			**Exp3**		
**Line**	**Area#**	Sig*	**Line**	**Area**	**Sig**	**Line**	**Area**	**Sig**
WT	21100	a	WT	35970	a	WT	17800	a
2G	44800	b				2G	107800	b
4C	56400	bcd	4C	71383	b	4C	123080	b
			3E	86030	bc	Tc	172943	c
2B	60400	cd	4E	98700	c	2B	195950	c
3E	69990	d	Tc	127150	d	4E	258350	d
2F	102477	d	2F	153100	e	2F	297140	d

Mutants were ranked in each experiment according to mean infection site area.

*Tukey test results for the pairwise differences between means in the analysis of the infection site areas. Means with the same letter within an experiment are not significantly different at P < 0.05.

# arbitrary units.

In summary, the leaf rust results from microscopic and macroscopic studies are in accordance with results obtained for stripe rust using field observations and fungal biomass quantification consistent with these mutants encoding heritable differences in their level of susceptibility to these pathogens. However, no detectable differences between mutants were observed in response to infection by stem rust.

### Relating phenotypic differences to mutations in DNA sequence

**Figure 3:**
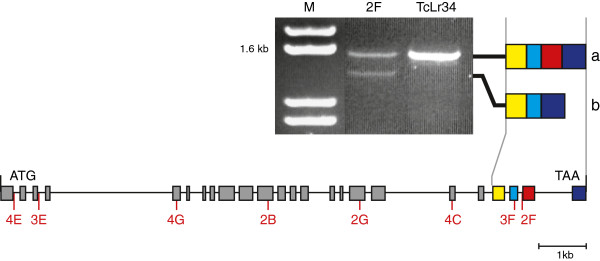
***Lr34 *gene structure with open boxes indicating exons and the position of mutations was marked.** RT-PCR of RNA extracted from flag leaf of mutant 2F amplified two transcripts. Sequencing of products confirmed the wild type *Lr34* transcript of 1,571 bp **(a)** and a mis-spliced product with exon 23 (255 bp) removed **(b)**[15].

**Figure 4:**
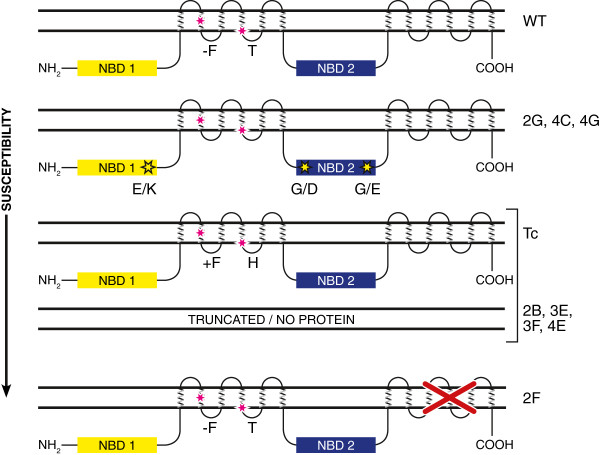
**Diagram of predicted Lr34 protein showing nucleotide binding domains NBD1 and NBD2 and two transmembrane domains.** The Lr34 protein differs from the protein encoded by the Thatcher allele by the absence of a phenylalanine residue (F) and a substitution of histidine (H) for tyrosine (T) in first transmembrane domain. Based on different responses to leaf rust and stripe rust infections, mutants were placed into three categories of susceptibility; partial susceptibility caused by single amino acid changes in the first or second NBD (mutants 2G, 4C and 4G), full susceptibility, similar to Thatcher, in which mutants were predicted to encode severely truncated and probably non-functional proteins (mutants 2B, 3E, 3F and 4E) and hyper-susceptibility (more susceptible than Thatcher) represented by mutant 2F which encoded a protein with a deletion of 85 amino acids in the second transmembrane domain.

Single nucleotide transition events (G to A or C to T), characteristic of sodium azide mutagenesis, were present in the *Lr34* putative ABC transporter gene in each of the eight mutants (Table 1 and Figure 3) [15]. Three categories of mutants were identified. Four mutants (2B, 3E, 3F and 4E) encoded nonsense mutations in the *Lr34* ORF and are predicted to produce truncated and probably non-functional proteins. The responses to stripe rust and leaf rust infection of these mutants were indistinguishable from the susceptible Thatcher parent. Three mutants (2G, 4C and 4G) encoded missense mutations that result in single changes within highly conserved amino acids of the predicted first or second nucleotide binding domain of the protein (Figure 4). All three mutants showed an intermediate accumulation of stripe rust chitin in flag leaves compared with the parental lines, while seedlings of mutants 2G and 4C were also partially susceptible to leaf rust. The hyper-susceptible mutant 2F encoded a mutation at the 3′ splice site of intron 22 resulting in transcripts encoding the predicted wild type protein and alternative transcripts lacking exon 23 (Figure 3), encoding a protein with part of the second transmembrane domain deleted (Figure 4). This mutant was more susceptible to stripe rust and accumulated the highest level of stripe rust biomass in flag leaves under field conditions, in addition to showing enhanced seedling susceptibility to leaf rust at the seedling stage.

### Isolation and characterisation of Lr67 mutants

**Figure 5:**
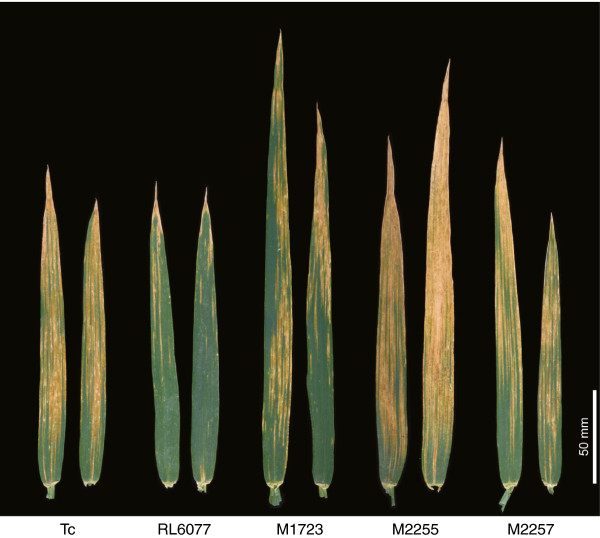
**Stripe rust infection on flag leaves of field grown of Thatcher (Tc, susceptible control), RL6077 (*Lr67*) and derived mutants M1723, M2255 and M2257.** M2255 was more susceptible to stripe rust than Thatcher.

Four stripe rust susceptible mutants were isolated from M2 families after treating RL6077 (*Lr67*) seed with sodium azide. These putative mutants were progeny tested in the field as M3 and M4 rows to confirm the loss of stripe rust resistance. M4 rows were scored at two field sites where mutant M2255 (70-80MS) was more susceptible to stripe rust than Thatcher (40-60MS) at both sites (Figure 5). Chitin quantification also showed that M2255 accumulated more stripe rust in flag leaves at anthesis than other mutants (Figure 1). The level of susceptibility of the other mutants, M1723, M2006 and M2257, was similar to Thatcher in the field although M2006 and M1723 contained less stripe rust in flag leaves than Thatcher.

**Figure 6:**
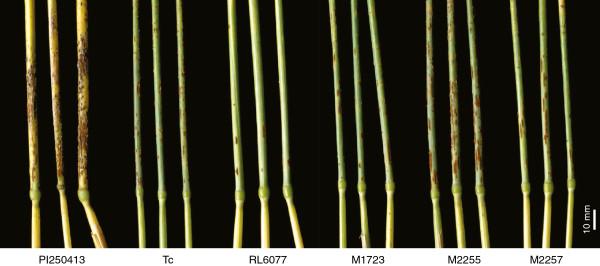
**Stem rust infection on the peduncle of field grown PI 250413 (donor *Lr67*), Thatcher (Tc), RL6077 (Lr67) and derived mutants M1723, M2255 and M2257.** M2255 was more susceptible to stem rust than Thatcher.

The effect of *Lr67* on resistance to stem rust was also recorded. RL6077 (5MR-10MR) produced fewer pustules under field conditions than the moderately resistant/susceptible Thatcher line (30–40 MRMS) (Figure 6). Mutants M1723, M2006 and M2257 were moderately resistant/susceptible and similar to Thatcher, but M2255, which was more susceptible to stripe rust, was also more susceptible to stem rust (60MS) than the other mutants (Figure 6). The *Lr67* donor line PI 250413 was fully susceptible to stem rust (90S) indicating that *Lr67* alone was insufficient to confer stem rust resistance and that the stem rust resistance present in the Thatcher backcross derivative, RL6077, was due to interaction of *Lr67* with other gene(s) present in this genotype. Lr67 mutants were also susceptible to leaf rust although the field infection was more variable than for stripe rust and stem rust. Several attempts to induce a resistance response to leaf rust in seedlings when grown at low temperature failed in RL6077. In contrast to RL6058, RL6077 was fully susceptible to leaf rust under these conditions. Leaf tip necrosis which was associated with *Lr67* was also lacking in these mutant lines.

### Comparison of Lr34 with Lr67

The resistance response of isogenic *Lr34* and *Lr67* lines to stripe rust and stem rust was similar, although in some field trials Lr34 resistance was marginally stronger. Previous studies also reported that field leaf rust resistance conferred by *Lr34* was more effective than *Lr67*[7,19]. The response to stripe rust infection revealed differences amongst both Lr34 and Lr67 mutants. Mutations within the *Lr34* gene produced partial, full and hyper-susceptible mutants. A hyper-susceptible mutant was also recovered amongst Lr67 mutants and the quantification of fungal biomass suggested that partially susceptible mutants may have also been found but their phenotype was indistinguishable from other mutants under field conditions. An important difference between *Lr34* and *Lr67* was the lack of seedling resistance to leaf rust in RL6077 when grown under low temperature conditions. Under field conditions, both genes confer resistance to stripe rust, leaf rust and stem rust. Independent Lr34 mutants provided proof that a putative ABC transporter gene was responsible [15]. Having been unable to separate the resistances to the different pathogens by mutation, we hypothesise that a single gene encoded by *Lr67* is also responsible for resistance to the three rust pathogens in RL6077.

## Discussion

Different mutations within the *Lr34* gene were associated with different levels of susceptibility to stripe rust and leaf rust. Mutants encoding single amino acid substitutions in the first or second nucleotide binding domain of the predicted protein showed only a partial loss of resistance suggesting that transport activity of the protein was compromised but not abolished, in contrast to fully susceptible mutants with predicted complete loss of protein function. Mutants that are expected to produce a severely truncated or non-functional protein were as susceptible as Thatcher, which encodes a predicted full length protein that varies by only two amino acid residues from Lr34 [15]. These data imply that the protein encoded by the Thatcher allele may be non-functional and does not confer a basal level of resistance required for normal plant growth and development. The hypothesis requires further investigation given the level of variability in infection between Thatcher and fully susceptible lines across experiments. The hyper-susceptible 2F mutant encodes a Lr34 protein containing a deletion in the second transmembrane domain which may act as a ‘dominant negative’ mutant that interferes with other related transporters that contribute to basal immunity.

Analysis of Lr34 and Lr67 mutants confirmed previous reports that these genes confer resistance to multiple pathogens in a race non-specific manner. Given that sodium azide produces point mutations, the mutation analysis of *Lr67* is consistent with a single gene conferring all resistance phenotypes associated with this locus. While the transfer of *Lr34* or *Lr67* into susceptible wheat backgrounds results in resistance to leaf rust and stripe rust, these genes do not confer effective stem rust resistance by themselves. For example, wheat lines containing *Lr34* such as Chinese Spring, Terenzio and Sumai 3 are susceptible to stem rust. However, transfer of *Lr34* into Thatcher results in the expression of effective stem rust resistance possibly through interaction of *Lr34* with unlinked Thatcher genes. There is good evidence that the ‘enhancement effect’ of *Lr34* on stem rust resistance is race specific suggesting that the mode of action is different to the race non-specific response conferred by this gene against leaf rust and stripe rust [11,12,14]. This study has confirmed that *Lr67* is also associated with enhanced stem rust resistance in Thatcher. The donor line of *Lr67*, PI 250413, was stem rust susceptible demonstrating that *Lr67* resembles the *Lr34* phenotype in that it also interacts with unlinked genes in Thatcher for expression of stem rust resistance [19]. It remains to be determined if the *Lr67* response to stem rust is also race specific and if it requires the same Thatcher genes as *Lr34* for resistance.

If these genes were combined, would they provide additional protection to three rust pathogens? A putative *Lr34*/*Lr67* line (90RN2491) which was previously developed and screened for rust resistance is unlikely to carry *Lr67* after this line was genotyped with a newly developed marker for *Lr67* [19, Spielmeyer, unpublished]. New germplasm will need to be developed to evaluate the resistance response of the gene combination. Given the phenotypic similarities, it is possible that *Lr34* and *Lr67* encode proteins with similar function and lines carrying gene combinations that encode functionally redundant proteins may not out-perform single gene lines. The comparison of phenotypic differences amongst Lr67 mutants to causal mutations awaits the isolation of the gene.

## Conclusion

Isolation and characterisation of mutants for *Lr67* and *Lr34* genes in a common genetic background enabled detailed studies of the resistance response to three rust pathogens. Mutants were assessed visually in the field, by quantifying chitin levels in field samples, macroscopic observations and microscopic analysis of seedlings grown under controlled conditions. The isolation of partial and hyper-susceptible mutants for both genes from within relatively small M2 populations is interesting and may provide insights how predicted proteins function and interact with components of basal resistance in future studies. We anticipate that marker development for *Lr67* and as yet unidentified Thatcher- derived gene(s) that interact with *Lr34* and *Lr67* to confer stem rust resistance will add further value to both APR genes and provide greater rust protection in future cultivars.

## Methods

### Plant material

Near-isogenic lines were developed by P. Dyck (AgCanada) by transferring *Lr34* from the Chinese landrace PI 58548 and *Lr67* from the Pakistani landrace PI 250413 by 6 backcross generations into the susceptible Thatcher background to produce RL6068 and RL6607, respectively.

### Rust infection and phenotyping under controlled conditions

To screen for leaf rust resistance under low temperature conditions, one week old seedlings were inoculated with *P. triticina* pathotype 104–1,2,3,(6),(7),11 and incubated for 24 hrs at 20°C and high humidity before being moved to a growth cabinet set at a 10°C constant temperature and 16 hrs photoperiod.

### Rust infection and phenotyping under field conditions

Lr34 mutants, parental lines and susceptible Thatcher control were grown in replicated field rows in a rust nursery at Cobbitty (Australia) in 2007, 2008, 2009 and 2012. Stripe rust *Pucciniia striiformis* f. sp. *tritici* pathotypes 134 E16 A + and 104 E137 A- +17, leaf rust *P. triticina* pathotypes 104–1,2,3,(6),(7),9,11; 104–1,2,3,(6),(7),11,13; 10–1,3,7,9,10,12; 76–3,5,9,10 + Lr37 and stem rust *P. graminis* f.sp. *tritici* pathotypes 98–1,2,3,5,6 and 34–1,2,7 + Sr38 were progressively released from tillering to late stem elongation stage. The modified Cobb scale was used to rate the disease responses with TR = trace resistant, MR = moderately resistant, MS = moderately susceptible and S = susceptible [20]. Lr67 mutants, parental lines and controls were progeny tested in replicated field rows in 2010 at Cobbitty where they were scored for stripe rust and leaf rust resistance. In 2011, Lr67 mutants were evaluated in replicated rows at Cobbitty for stripe rust, leaf rust and stem rust resistance and again for stripe rust resistance (natural infection) in an experimental site at Canberra, Australia. In 2012, Lr67 mutants were pathotyped again at two sites in Cobbitty, Australia.

### Isolation of mutants

Seed of RL6058 and RL6077 was pre-soaked in water for 12 hrs at 4°C before treating the grains in an oxygenated solution of 7 mM sodium azide at pH 3.0 for 2 hrs. The grains were rinsed and planted in the field. For each parental line approximately 1000 M2 single ear rows were scored for stripe rust resistance under field conditions. Seed was collected from putative mutants and resistant sibs and progeny plants of each were tested over several seasons for stripe rust, leaf rust and stem rust resistance in rust nurseries at Cobbitty and Canberra, Australia.

### Microsopy and measurements of rust infection site areas

Wheat leaves (mostly 1^st^ leaf) were inoculated with leaf rust and after 10 days prepared and stained with WGA-FITC as described in [18]. Infection site areas which had produced infection hyphae were photographed under blue light using x10 magnification. Approx. 30–40 randomly selected infection sites were photographed for each line in one experiment and infection site areas calculated from photographic images using the AnalySIS Life Science Professional program (Olympus, Mt. Waverley, Australia). Kruskal-Wallis one way analysis of variance and Tukey test for the pairwise differences between means was used for statistical analysis of the data.

### Wheat germ Agglutinin Chitin (WAC) assay

Flag leaves were harvested from 20–30 plants, at or near anthesis, grown in replicated field rows that were inoculated with stripe rust (see above). Tissue was cut into approx 2 cm long segments and mixed together before sub-sampling 1–2 grams of tissue for the chitin assay. Chitin quantification was undertaken as described in [16]. Briefly, tissue segments were autoclaved in 1 M KOH and then neutralised in 50 mM Tris pH 7.0. The tissue was then homogenised by sonication and resuspended at a final concentration of 200 mg/ml. Replicated 20 mg samples were stained with WGA-FITC and tissues then washed three times with 50 mM Tris pH 7.0 buffer. Samples were finally resuspend in 100ul and fluorescence measured using a Wallac Victor 1420 multilabel counter (Perkin and Elmer Life Science) fluorometer with 485 nm adsorption and 535 nm emission wavelengths and a 1.0 second measurement time. A minimum of three replicates were undertaken for all samples.

## Competing interests

There were no financial or non-financial competing interests identified.

## Authors’ contribution

WS conceived and designed the experiments. RM and CW contributed to the isolation of mutants. MA developed the method for measuring rust infection sites and developed and carried out the chitin assay. All authors read and approved the final manuscript.
